# Dual role of the *Anopheles coluzzii* Venus Kinase Receptor in both larval growth and immunity

**DOI:** 10.1038/s41598-019-40407-x

**Published:** 2019-03-05

**Authors:** Nadège Gouignard, Floriane Cherrier, Emma Brito-Fravallo, Adrien Pain, Natalia Marta Zmarlak, Katia Cailliau, Corinne Genève, Kenneth D. Vernick, Colette Dissous, Christian Mitri

**Affiliations:** 1 CIIL- Institut Biologie de Lille, Inserm U1019, CNRS UMR 8204, Institut Pasteur Lille, Lille, France; 2 Genetics and Genomics of Insect Vectors Unit, Department of Parasites and Insect Vectors, Institut Pasteur, Paris, France; 3 Centre National de la Recherche Scientifique, UMR2000 Paris, France; 4 Present Address: Oncogenesis of Lymphoma unit, INSERM U1053 - BaRITOn, Bordeaux, France; 5 Present Address: Department of Basic Science & Craniofacial Biology, New York University, College of Dentistry, New York, USA; 6 Institut Pasteur – Bioinformatics and Biostatistics Hub – C3BI, USR, 3756 IP CNRS Paris, France; 7 Team “Signal Division Regulation”, CNRS UMR 8576, University of Lille, Lille, France

## Abstract

Vector-borne diseases and especially malaria are responsible for more than half million deaths annually. The increase of insecticide resistance in wild populations of *Anopheles* malaria vectors emphasises the need for novel vector control strategies as well as for identifying novel vector targets. Venus kinase receptors (VKRs) constitute a Receptor Tyrosine Kinase (RTK) family only found in invertebrates. In this study we functionally characterized *Anopheles* VKR in the Gambiae complex member, *Anopheles* *coluzzii*. Results showed that *Anopheles* VKR can be activated by L-amino acids, with L-arginine as the most potent agonist. VKR was not required for the fecundity of *A. coluzzii*, in contrast to reports from other insects, but VKR function is required in both *Anopheles* males and females for development of larval progeny. *Anopheles* VKR function is also required for protection against infection by *Plasmodium* parasites, thus identifying a novel linkage between reproduction and immunity in *Anopheles*. The insect specificity of VKRs as well as the essential function for reproduction and immunity suggest that *Anopheles* VKR could be a potentially druggable target for novel vector control strategies.

## Introduction

Vector-borne diseases account for more than 17% of all infectious diseases, causing more than 700,000 deaths annually^1^. Malaria, the most deadly vector-borne disease still causes about 500,000 deaths annually in the world^2^. This disease is caused by the Apicomplexa parasite of the genus *Plasmodium*, and is transmitted to humans by an infective bite of *Anopheles* mosquitoes. Most of malaria cases and deaths occur in sub-Saharan Africa, where mosquitoes from the *Anopheles gambiae* sensu lato complex constitute the main vectors of *Plasmodium* parasites. Vector-targeted strategies accounted for 80% of the gains in malaria reduction in Africa in recent decades^3^, and this will very likely remain the case in future. However, with the increase of insecticide resistance in the field^4–6^, new vector control strategies are needed, and a better knowledge of the malaria vector system and of the mosquito biology are thus strongly required.

Venus Kinase Receptors (VKRs) constitute a Receptor Tyrosine Kinase (RTK) family, originally found in the parasite platyhelminth *Schistosoma mansoni*^7^, then in a large number of invertebrate species, and particularly in many insects^8^. However, VKRs were not found in any vertebrate organism and strikingly, they are not present in the nematode *C. elegans* nor in the fruit fly *Drosophila melanogaste*r^9^. VKR proteins possess an atypical structure^7,9^, with an intracellular tyrosine kinase (TK) domain similar to that of insulin receptors (IR) and an extracellular Venus Flytrap (VFT) domain. VFTs were first identified as bacterial periplasmic-binding proteins involved in the transport of small molecules, such as amino acids, sugars or ions and they constitute the ligand binding pocket of different receptor types such as class C G-protein coupled receptors^10^. Sequence alignments of the VFT domains from various VKRs revealed that they contain the signature sequence required for L-AA binding^11^ and previous results showed that several VKRs are effectively activated following the binding of L-AA to the extracellular VFT domain^9,12^.

Transcripts of *vkr* were found to be abundant in larval forms and in female ovaries of the platyhelminth *S. mansoni*^13^ and of several insects like *Tribolium castaneum, Apis mellifera, Anopheles gambiae*^9^. They were found in the ovaries of *Aedes aegypti*^14^ and more recently in the gonads of the desert locust, *Schistocerca gregaria*^15^. Functional studies using RNAi-mediated genes silencing showed that VKRs control reproduction, and especially the production of eggs in *S. mansoni*^13^ as well as in the mosquito vector *A. aegypti* in which VKR was demonstrated to be a receptor for the ovary ecdysteroidogenic hormone (OEH)^14^. VKRs were also shown to control testis development and spermatogenesis in *S. mansoni* male parasites^13^. Unlike in *A. aegypti* and *S. mansoni*, VKR does not seem to be essential for reproduction in *S. gregaria*, since silencing of SgVKR did not affect fecundity or fertility^15^.

Here we performed the functional characterization of *Anopheles* VKR in *Anopheles coluzzii*, a member of the Gambiae species complex of major malaria vectors^16^. We cloned the VKR gene from an *A. coluzzi* laboratory strain and expressed the protein in stage VI *Xenopus* oocytes. These cells are blocked in the first period of meiosis (metaphase 1) but activation of RTK can induce further kinase signalling cascades and Akt and MAPK phosphorylation can result in meiosis resumption and Germinal Vesical BreakDown (GVBD)^17^. In this heterologous system, we demonstrated the TK activity of *A. coluzzii* VKR and identified L-Arginine as its most potent activating ligand. In addition to expression in the mosquito gonads, we also found that *vkr* transcripts were expressed in *Anopheles* haemocytes, which are immune-competent cells. Functional studies using RNAi-mediated gene silencing revealed a dual role for the *A. coluzzii* VKR in both the reproduction of this insect, as well as immunity against infection with malaria parasites.

## Materials and Methods

### Cloning of *Anopheles* VKR

The VKR of *A. gambiae* M (now named *A. coluzzii*) was identified by screening the available expressed sequence tag (EST) and genomic databases (Flybase: http://flybase.org/)) with the protein sequence of SmVKR1 from *Schistosoma mansoni*^7^ using the TBLASTN program. The complete cDNA sequence of *A. coluzzii* VKR was determined by 5′ and 3′ RACE amplification (GeneRacer® Kit, Invitrogen) according to the manufacturer’s instructions. Fragments encompassing the complete coding sequence of VKR were obtained by PCR on adult mosquito cDNA using Advantage 2 Polymerase mix (Clontech Laboratories, Inc.) and the following primer sequences, AgVKRflF (5′-CCGTCTGTGCCCGTGGATCACTGCG-3′) and AgVKRflR (5′-GCTGTGCAGTGGCAAGGTGACGGATC-3′). PCR products were purified from agarose gels using the extraction kit Wizard^®^ SV Gel and PCR clean-up system (Promega) and inserted into pCR2.1-TOPO (Invitrogen). Selected clones of full length VKR were sequenced by GATC Biotech. The *A. coluzzii* VKR nucleotide sequence was previously deposited in Genbank under the name AgVKR with the accession number EU878397.1^9^.

### VKR constructs and site-directed mutagenesis

AgVKR cDNA sequence was subcloned into the mammalian expression vector pCDNA3.1-V5-His (Invitrogen) (VKR^WT^) by an in frame insertion using EcoRI and NotI sites. Site-directed mutagenesis was used to change the Serine 505 to Alanine in the VFT domain of VKR (VKR^S505A^) using the QuikChange Site–Directed Mutagenesis Kit (Stratagene). The 5′-CTGGGTCCTGCCTGC*gc*TGAAACGGTCGAACCAATTGC-3′ mutated sequence and its reverse complement were used as primers to mutate Serine 505 into Alanine (mutated residues are in lowercase italics).

### Expression of recombinant VKR in *Xenopus* oocytes

cRNAs encoding VKR^WT^ or VKR^S505A^ proteins were synthesized *in vitro* from plasmids previously linearised by PmeI enzyme using the T7 mMessage mMachine Kit (ambion, USA). cRNA transcribed from 1 µg of each linearised plasmid was precipitated by 2,5 M LiCl, washed in 70% ethanol, resuspended in 20 µl diethylpyrocarbonate (DEPC)-treated water, then quantified by spectrophotometry. Finally, 1 µg of cRNA was analysed on a denaturating agarose gel. Gel staining with 10 µg/ml ethidium bromide allowed confirmation of the correct size of cRNA and verification of the absence of abortive transcripts. 60 nL of cRNA preparations (1 mg/ml) were microinjected in *Xenopus laevis* oocytes in the equatorial region according to the protocol previously described^18^ and incubated for 18 h at 19 °C in ND96 medium (96 mM NaCl, 2 mM KCl, 1 mM MgCl_2_, 1.8 mM CaCl_2_, 5 mM Hepes pH 7.4 supplemented with 50 µg ml^−1^ streptomycin/penicillin, 225 µg ml^−1^ sodium pyruvate, 30 µg ml^−1^ trypsin inhibitor) supplemented or not with L-amino acids and/or kinase inhibitors in order to evaluate VKR kinase activation and induction of germinal vesicle breakdown (GVBD) in oocytes, a process easily detectable by the appearance of a white spot at the centre of the animal pole of the oocyte. Expression of VKR proteins in oocytes was confirmed by immunoprecipitation of membrane extracts according to the procedure described previously^18^ using anti-V5 antibodies (1:100, Invitrogen). Immune complexes were analysed by Western blotting using anti-V5 (1:50 000) or PY20 (1:10 000, antiphosphotyrosine, BD Biosciences) antibodies and the advanced ECL detection system (Amersham Biosciences).

### Nucleotide and protein sequence analyses

Sequence analyses were performed using the LASERGENE package (DNAStar, Madison, WI, USA). The AgVKR cDNA was used to determine the genomic structure using BLAST analysis (http://flybase.org/blast/) on FlyBase data bank. The exon-intron boundaries were slightly modified by eye. The signal peptide, the VFTM and TK domains were delimited by SignalP 3.0 server (http://www.cbs.dtu.dk/services/SignalP/), InterProScan(http://www.ebi.ac.uk/Tools/InterProScan/) and BLAST structural analysis algorithms. Motif research was made using ELM server (Eukaryotic Linear Motif: http://elm.eu.org). Pair-wise mannered alignments were generated with Clustal W program (MEGΛ4).

### RNA interference

The N’gousso mosquito colony, initiated in Cameroon in 2006, then implemented in 2007 in the CEnter for Production and Infection of Anopheles (CEPIA) at Pasteur Institute, was used for functional tests. The colony, previously described as *A. gambiae* M molecular form, is *A. coluzzii* in the current nomenclature. Gene specific fragments of *Anopheles* VKR and GFP (dsRNA control) were generated from *A. coluzzii* cDNA or GFP-containing plasmids by PCR using T7 promoter-tagged primer sequences (T7-VKR-F/T7-VKR-R and T7-GFP-F/T7-GFP-R respectively; Table S2). PCR products were used as templates for *in vitro* dsRNA synthesis using the MEGAscript RNAi Kit (Invitrogen). 500 ng of dsRNA were injected into the thorax of ice-anesthetized 1–2 days old females or 4 days old males *A. coluzzii* using a nanoinjector (Nanoject II; Drummond Scientific). Four days after the dsRNA treatment, knockdown efficiency of the *Anopheles* VKR gene was checked using total RNA from pools of five injected mosquitoes. Using 1μg of total RNA, reverse transcription followed by quantitative PCR was performed with *Anopheles* VKR specific primers (VKR-q-F and VKR-q-R; Table S2). The same samples were also used to measure the expression level of the CYP307A and CYP314A, both involved in the biosynthesis of the 20-Hydroxyecdysone. Ribosomal gene rpS7 was used as an internal calibrator for normalisation. Specific primers for transcript quantification of CYP307A (CYP307-q-F and CYP307-q-R), CYP314A (CYP314-q-F and CYP314-q-R) and of the internal calibrator rpS7 (rpS7-qF and rpS7-qR) are listed in Table S2. Analysis of the expression of transcripts relative to rpS7 was performed according to the 2−ΔΔCt method^19^. Difference in delatCt distribution between dsGFP and dsVKR was statistically tested using Wilcoxon signed rank non-parametric test.

### *Plasmodium* Infection and oocyst detection

Females *Anopheles* previously injected with dsGFP (control group) or dsVKR were fed on mice infected with *P. berghei* strain PbGFPCON^20^, which constitutively expresses green fluorescent protein (GFP) and produces mature gametocytes. After the infectious blood meal, unfed mosquitoes were removed and fully engorged females were maintained at 21 °C and 70% relative humidity on 10% sucrose.

Mosquito midguts were dissected at 8 days post-infection, and the number of oocysts (the *Plasmodium* parasite stage located in the mosquito midgut) was counted by fluorescence microscopy. A mosquito was considered infected if at least one oocyst is seen in its midgut. Prevalence of infection is defined as the proportion of infected mosquitoes among the total dissected mosquitoes. For each gene-silencing experiment at least 25 surviving mosquitoes were analyzed for oocyst detection. For statistical analysis, differences in infection prevalence were tested using Chi Square. Following independent statistical tests for each replicate, and when the direction of change of each independent replicate was concordant, the p-values from independent tests of significance were statistically combined using the meta-analytical approach of Fisher^21^.

### *Anopheles* female ovary size and structure and number of developed oocytes

4 days post dsRNA injection (dsGFP or dsVKR), *Anopheles* females were allowed to feed on naive mouse for inducing vitellogenesis and eggs production. Dissection of individual female was performed at 48 h post-blood meal to collect their ovaries. The ovary size was measured under a light microscope (20× objective), where pictures were taken for each female ovaries from control dGFP and dsVKR groups. A similar experiment was made, but the pictures of the ovary pairs were used to count the number of developed oocytes in each ovary pair. Differences in ovary sizes or in the number of developed oocytes between the two groups were tested.

### Effect of VKR on fecundity and on progeny larval growth

#### Females (mothers) injection

Injections of dsGFP or dsVKR were performed in 1–2 days old virgin *A. coluzzii* females of. 3 days post injection the injected females were put in contact with 1-week-old non-injected males *A. coluzzii* in a 1:3 ratio (1 male for 3 females). 6 days post-injection these females were allowed to take a blood meal on naïve mouse and were maintained at 25 °C, 75% humidity. Egg laying was assessed for individual females and for pools of 40 females. For the latter case, 72 h post-blood feeding, the number of laid eggs was counted and reported to the number of females (40 females mothers) as a proportion and the difference of this proportion between dsGFP and dsVKR groups was tested using Chi Square. For the eggs laid from the individual females, the number of eggs was counted individually and differences between the two groups were tested using non-parametric Wilcoxon Mann-Whitney.

For the larval growth, 72 h post-feeding the eggs are collected from the two groups of females (dsGFP and dsVKR), counted, put in water pans and stored at 26 °C for larval growth. The number of emerging adults from each pan (dsGFP or dsVKR) was counted and reported to the number of initial laid eggs, as a proportion. The difference of this proportion between dsGFP and dsVKR groups was tested using Chi Square.

#### Males (fathers) injection

Injections of dsGFP or dsVKR were performed on 4 days old virgin *A. coluzzii* males. In order to let them release their stock of spermatozoids acquired during their larval development, and to allow a renewal of spermatozoids under VKRkd background, the 2 groups of males were allowed to mate with a first group of virgin females, 2 days post-injection in a 1:3 ratio (1 male for 3 females). Then, 3 days later, the 2 groups of injected males were all collected and put in contact with a new batch of virgin females for 2–3 days (still in a 1:3 ratio). These second batch of virgin females mated with dsGFP or dsVKR males were allowed to feed on naïve mouse and were maintained at 25 °C, 75% humidity. As described above, eggs were collected 72 h post-feeding, put in water and allowed to hatch and become adult. As for the injected females, the proportion of emerging adults was compared between dsGFP or dsVKR groups using Chi Square.

### Haemocyte collection from adult *Anopheles* females

Haemocytes were collected by perfusion from 20 *Anopheles* mosquito females as described in^22^. Basically, the penultimate abdominal segment of each mosquito was cut and intrathoracic perfusion with an anticoagulant buffer (60% Schneider’s medium, 10% FBS, 30% citrate buffer [98 mM NaOH, 186 mM NaCl, 1.7 mM EDTA, 41 mM citrate]) was performed. Using a Hamilton syringe system, ~10 µl of perfused haemocoel was collected from each perfused mosquito on tubes stuck into ice. The collected haemolymph from the 20 mosquitoes was pooled and centrifuged at 1,500 rpm for 5 min at 4 °C in order to collect the haemocytes. The buffer was removed and TRIzol reagent (Invitrogen) was added to resuspend the haemocytes and performed a total RNA extraction.

### Ethical and regulatory considerations

This study was conducted in strict accordance with the recommendations from the Guide for the Care and Use of Laboratory Animals of the European Union (European Directive 2010/63/UE) and the French Government. All procedures were approved by the Hygiene and Security Commission of the Institut Pasteur (protocol CHSCT N°13.313). The experiments on *Xenopus laevis* were carried in accordance with the principles of the European Community Council recommendations (86/609/EEC). The protocols were approved by the “Comité d’Ethique en Expérimentation Animale, Hauts de France”(CEEA, 07/2010). All methods were performed in accordance with the relevant guidelines and regulations.

## Results

### Cloning of *A. coluzzii* VKR

**Figure 1:**
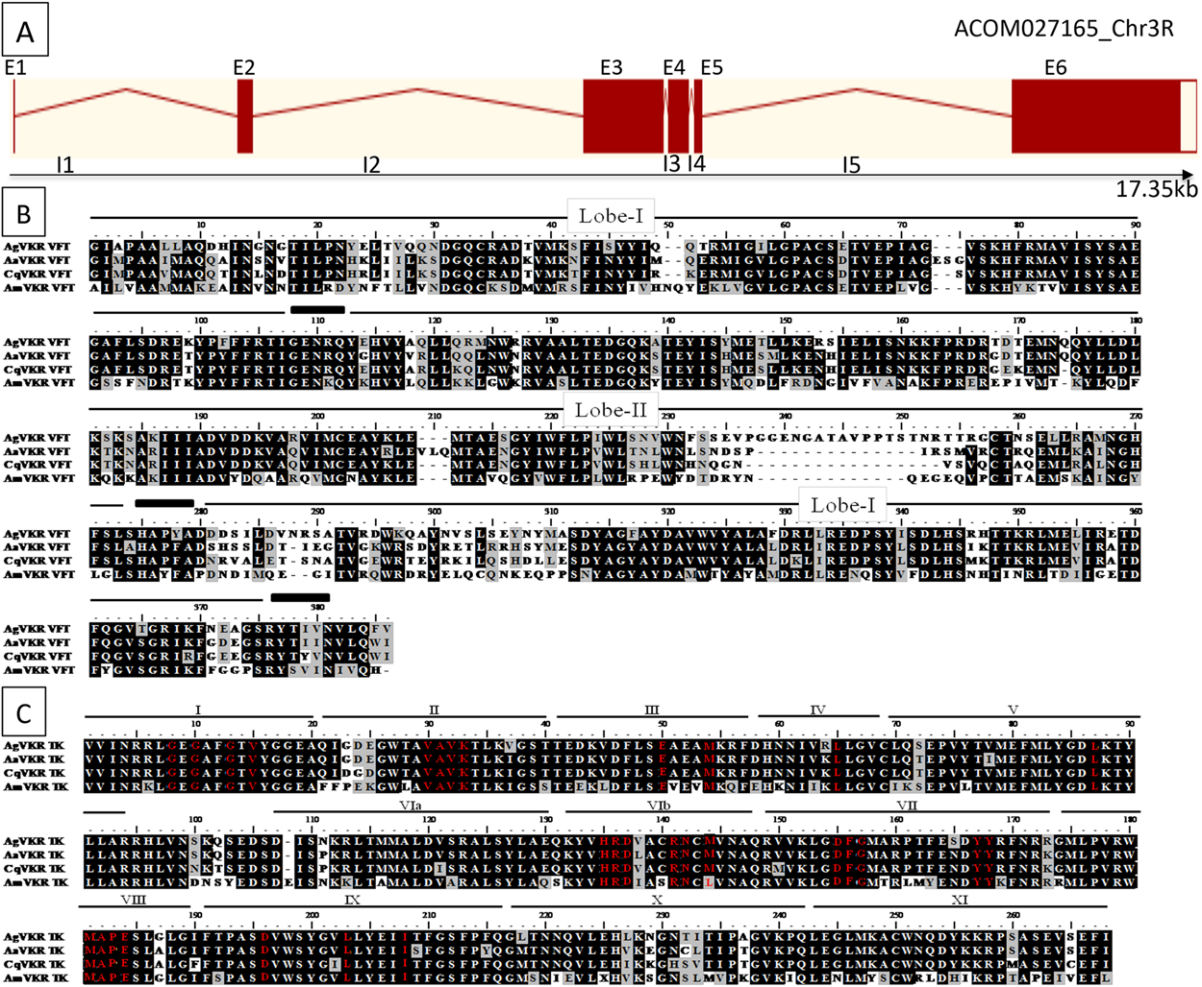
Genomic and protein structures of *Anopheles* VKR. (**A**) Exon-intron structure of VKR locus. Exons are represented as red boxes and introns as red lines. The arrow indicates the positions of the translational start (ATG) and stop (TAG) codons. The scale indicates the length in kb. (**B**) Protein sequence alignment of VFT from mosquitoes VKR (AgVKR ACF34410.1, CqVKR DAA06510.1, AaVKR DAA06509.1) and honey bee (AmVKR ACF34409.1) using the CLUSTAL W method. Lobe I and Lobe II (indicated by the upper black line) and the three linkers (black boxes) constitute the structure of VFT domains. (**C**) Sequence alignment of kinase domains of AgVKR, CqVKR, AaVKR and AmVKR. Numbers I to XI indicate the eleven subdomains conserved in protein kinase domains. Consensus sequences required for tyrosine kinase activity are indicated in red.

The reference genome assembly of *A. gambiae* was screened with a TBLASTN program to identify sequences related to the *S. mansoni* VKR sequence (SmVKR1). One EST sequence was identified as AGAP009158. The *vkr* gene is composed of six exons and five introns and is located in chromosome 3 R (Fig. 1A). The corresponding *A. coluzzii* gene (ACOM027165) was shown to display the same genomic organization. Using classic and RACE-PCR strategies, we cloned the complete cDNA sequence of VKR expressed by mosquitoes, which contains 5527 bp with a C terminal poly A tail. The sequence was submitted to Genbank under the name AgVKR (accession number EU878397)^9^.

### Comparative sequence analyses of *Anopheles* VKR with other insect VKR proteins

Amino-acid sequences of the VFT and TK domains of *Anopheles* VKR were aligned with sequences of VKR from two other mosquitoes, *Aedes aegypti* (AaVKR) and *Culex pipiens quinquefasciatus* (CqVKR) and from the honeybee *Apis mellifera* (AmVKR) (Fig. 1B,C). The multiple alignment showed that the *Anopheles* VKR sequence is more closely related to those of mosquitoes (*Aedes* and *Culex*) (65% identity) than to bees (*Apis*) (45% identity) in the VFT domain. Sequence analysis of *Anopheles* VKR confirmed the typical structure of its VFT domain formed by two lobes and three linkers that confer flexibility for the open-close conformation of the VFT domain (Fig. 1B). Our previous studies have shown that SmVKR1 and AmVKR possess a partially conserved sequence consensus for amino-acid binding and this is also the case for *Anopheles* VKR.

The TK domain of *Anopheles* VKR is also more similar to other mosquito (92%) than to bee VKRs (75%) (Fig. 1C). Like the other VKRs*, Anopheles* VKR possesses the eleven subdomains present in all protein kinases and the characteristic motifs essential for tyrosine kinase activity^9,12^. The GXGXXG motif in subdomain I is responsible for ATP binding and the VAVKX_16_E sequence (Subdomains II and III) is required for ATP stabilization. In subdomain VIb, the HRDXAXRNC sequence is implicated in the phosphotransfer on tyrosine residues. The DFG motif essential for the binding of Mg^2+^ is present in domain VII together with the two tyrosine residues, which are the site of receptor autophosphorylation in several RTKs including insulin receptors. Finally the PVRWMAPE sequence responsible for the conformation domain stabilization is present in subdomain VIII.

### L-Amino Acids (AA) activate *Anopheles* VKR through its VFT domain

We have previously shown that schistosome SmVKR could be expressed efficiently in *Xenopus* oocytes following injection of *in vitro* transcribed and capped messenger RNA^12,10^ and that several VKRs are effectively activated by L-AA^9^. The presence of the signature for L-AA binding in *Anopheles* VKR suggested that it could also be activated by these ligands.

**Figure 2:**
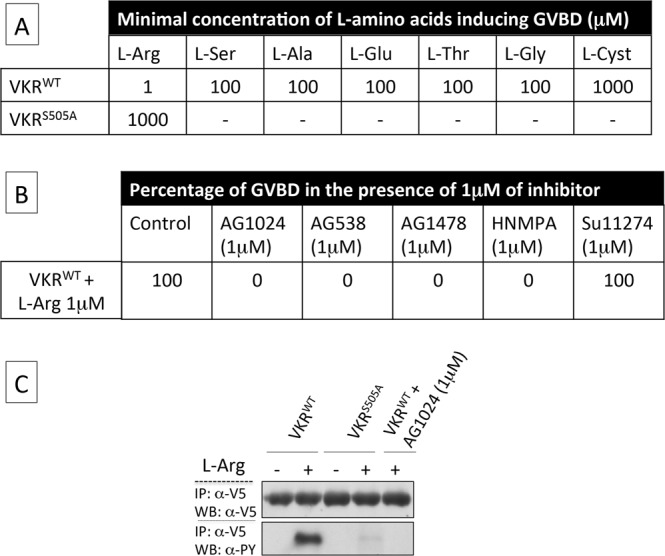
Functional characterization of *Anopheles* VKR in *Xenopus* oocytes. (**A**) Minimal L-AA concentrations to induce GVBD in *Xenopus* oocytes expressing *Anopheles* VKR (from 1 µM to 1 mM). Arginine is the most efficient L-AA to induce VKR activation. Mutation of Ser_505_ present inside the ligand binding pocket strongly decreases the capacity of VKR to be activated by L-AA. (**B**) Effect of various tyrosine kinase inhibitors on VKR activity induced by arginine binding evaluated by the percentage of GVBD in oocytes expressing VKR. AG1024, AG538 and HNMPA (IR inhibitors) and AG1478 (IR and EGFR inhibitor) totally inhibit *Anopheles* VKR activation at 1 µM whereas SU11274 (Met receptor inhibitor) has no effect at this concentration. (**C**) Native *Anopheles* VKR^WT^ or mutant VKR^S505A^ expressed in oocytes were immunoprecipitated from membrane extracts of oocytes using anti-V5 antibodies and revealed by Western Blot using anti-V5 and anti-PY antibodies for detecting tyrosine phosphorylation. VKR^WT^ is found to be phosphorylated in the presence of the ligand arginine but not in the presence of the inhibitor AG1024. The autophosphorylating activity of the mutant VKR^S505A^ is strongly affected.

It has been shown previously that expression and activation of VKR kinase induced germinal vesicle breakdown (GVBD) in *Xenopus* oocytes^12^. In this work, *Xenopus* oocytes were injected with cRNA encoding wild-type *Anopheles* VKR and we measured the capacity of the 20 classical L-AA to activate the VKR kinase and to induce GVBD. Results shown in Fig. 2A indicate that 7 L-AA (L-Arg, L-Ser, L-Ala, L-Glu, L-Thr, L-Gly and L-Cys) can induce GVBD and that L-Arg is the most potent agonist, already active at 1 µM whereas L-Cys is the least active, with a 1 mM concentration required (Fig. 2A). We also tested D-arginine and L-canavanine, which is structurally related to L-arginine, and we observed that both molecules were unable to activate *Anopheles* VKR (not shown).

*Anopheles* VKR possesses the strictly conserved serine residue (S_505_) already shown to be involved in the binding of the carboxylic group of α-AA in VFT modules^23^ and previously demonstrated to be required for the binding of L-AA by schistosome VKR^13^. Similar experiments of VKR activation by L-AA were performed using a mutant version of *Anopheles* VKR in which the S_505_ was replaced by an alanine residue. Results showed that L-AA were no more able to trigger GVBD in *Xenopus* oocytes expressing the mutant VKR^S505A^, except L-Arg, for which a 1 mM concentration was in this case necessary to induce GVBD (Fig. 2A), indicating the importance of S_505_ for ligand binding and activation of the *Anopheles* VKR. Western blot results (Fig. 2C) confirmed the expression of recombinant V5-tagged VKR at the oocyte membranes and the tyrosine phosphorylation of VKR in the presence of L-Arg. The inactive mutant VKR^S505A^ is no more highly phosphorylated in the presence of L-Arg, and this indicates that a functional VFT domain is required for *Anopheles* VKR activation by L-AA.

### *Anopheles* VKR is sensitive to insulin receptor kinase (IR) inhibitors

We further tested the sensitivity of the TK domain of *Anopheles* VKR to different tyrosine kinase inhibitors and evaluated the capacity of the tyrphostins AG1024, AG538, HNMPA (three IR inhibitors), AG1418 (IR/EGFR inhibitor) and SU11274 (Met inhibitor) to inhibit oocyte GVBD induced by L-Arg. At 1 µM, AG1024, AG538, AG1418, and HNMPA inhibited totally GVBD, whereas SU11274 had no effect (Fig. 2B). This result confirmed the similarity of the TK domain of VKRs with IR receptors^9^. Western blot analysis (Fig. 2C) confirmed the absence of tyrosine phosphorylation of L-Arg-bound VKR in the presence of 1 µM AG1024.

### VKR silencing did not affect ovary structure and fecundity in *A. coluzzii*

VKR transcripts have been already shown to be abundant in *Anopheles* gonads^9^. We therefore assessed whether VKR could play a role in *Anopheles* reproduction. To this aim, RNAi-mediated gene silencing was performed in two groups of two day-old virgin *Anopheles* females by intra-thoracic injection of dsRNA. One control group was injected with dsGFP and the other group with dsVKR. At three days post-injection, the females were allowed to mate with 7 day-old virgin males and were blood fed at day 6 post-injection.

**Figure 3:**
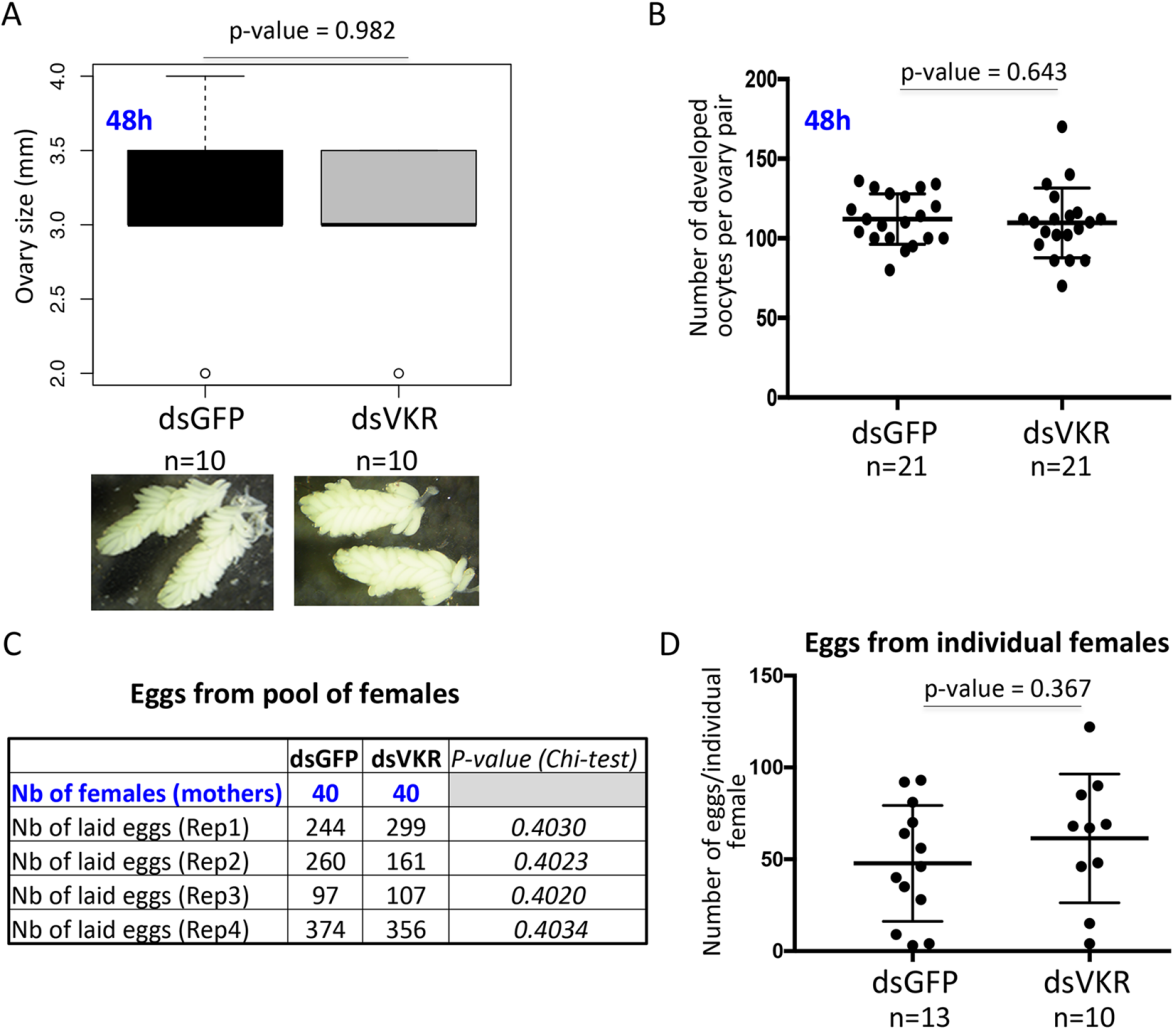
VKR does not affect *A. coluzzii* ovary structure and fecundity. (**A**) Median size and structure of *A. coluzzii* ovaries from dsGFP and dsVKR females at 48 h post-blood meal are not significantly different. (**B**) Median numbers of developed oocytes per ovary pair from dsGFP and dsVKR females at 48 h post-blood meal are not significantly different. The Differences in ovary sizes and in the number of developed oocytes between the two groups were tested using non-parametric Wilcoxon Mann-Whitney. (**C**) Eggs from pools of females: VKR has no effect on the number (Nb) of laid eggs reported to the number (Nb) of females (mothers) in four independent experiments (Chi-square p-value > 0.05). The number of female mothers for each group (dsGFP and dsVKR) is the same (40 females) for each biological replicate. (**D**) Eggs from individual female: VKR has no effect on the number of laid eggs per individual female (non-parametric Wilcoxon Mann-Whitney, p-value > 0.05).

Comparing the size and the structure of ovaries at 48 h post-blood feeding, we could not find any statistically significant difference between dsGFP and dsVKR groups (Fig. 3A). We also counted the number of developed oocytes in the ovary pair at 48 h post-blood feeding and we did not find a statistical difference between the two groups of females (Fig. 3B). To strengthen this observation, we checked the female fecundity in both dsGFP and dsVKR groups by counting the number of laid eggs from both pool of females and from individual female. From the pool of females, results from four independent experiments presented in Fig. 3C showed no difference in the number of laid eggs/female between dsGFP and dsVKR females. Similarly, looking at the number of eggs from individual females did not show statistical difference between dsGFP and dsVKR groups (Fig. 3D). This indicates that VKR is not required for *A. coluzzii* fecundity.

### VKR is required for *Anopheles* larval development

**Figure 4:**
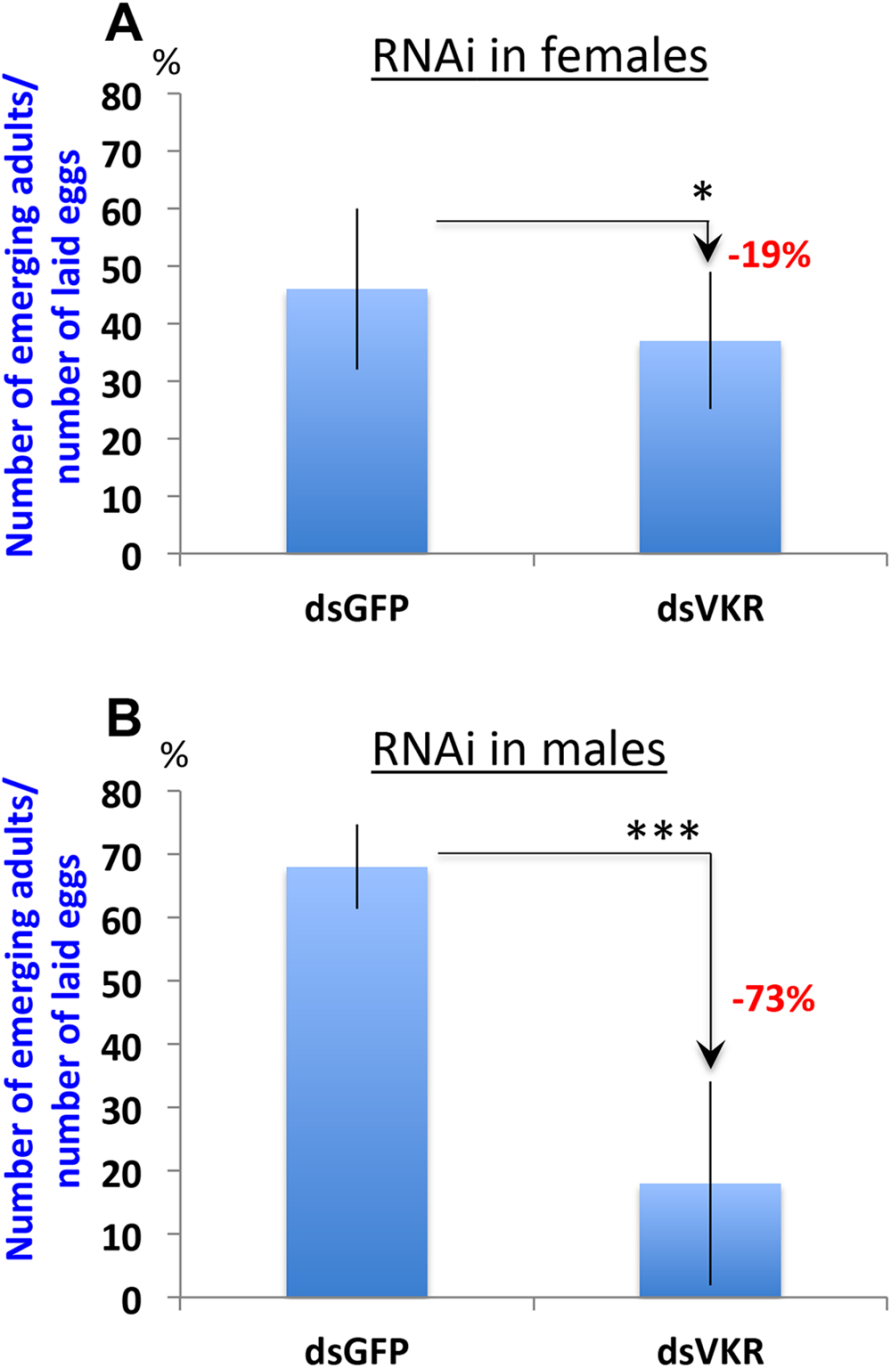
VKR is required in larval growth. VKR silencing either in female (**A**) or in male (**B**) mosquitoes affects the larval development of their progenies, considering the proportion of emerging adults reported to the number of eggs put in water. Stars (*) and (***) represent Chi2 test p-value < 0.05 and p-value < 0.001 respectively.

As VKR silencing in females did not affect their fecundity (Fig. 3), we further assessed the capacity of laid eggs to develop from larvae to adults. Silencing was performed in the same experimental conditions as above in two groups of *Anopheles* females (dsGFP and dsVKR). Eggs from each group were collected at 72 h post-blood feeding and placed in water. As no difference was found for the hatching rate between eggs collected from dsGFP and dsVKR female mothers (Supplementary Fig. S1), we then assessed the efficiency of these eggs to develop from larvae to adult stages. Three independent biological experiments were performed which showed a statistically significant reduction (19%, p = 0.044) of the proportion of emerging adults reported to the number of eggs put in water in VKR-silenced females (Fig. 4A).

As VKRs were previously considered to have functions in schistosome spermatogenesis^12^, we also assessed the role of VKR in offspring of males. dsRNA injections were performed in two groups of 4-day old *Anopheles* males (dsGFP and dsVKR), assuming VKR might be required for a good quality of spermatozoids. Therefore in order to test VKR function in male gonads, spermatozoid renewal should occur under dsVKR backgrounds. To this aim, two days post-dsRNA injection, the 2 groups of males were allowed to mate with a first group of virgin females in order to make them releasing their stock of spermatozoids acquired during their development. After mating with this first group of virgin females, the 2 groups of males (dsGFP and dsVKR) were put with a new group of virgin females during 3 days. This second group of mated females were blood fed in order to collect their eggs 72 h post-feeding. As it was observed in VKR-silenced females, we did not see any difference in the number of laid eggs from females mated with dsGFP or with dsVKR males (Supplementary Table S1). However, the development of these eggs from larvae to adult stage was strongly affected in the group of eggs issued from VKR-silenced fathers (p = 1e-05), since we observed more than 70% decreased survival in this larval group (Fig. 4B). This stronger phenotype observed when VKR is silenced in males as compared to females might be due to a more efficient silencing of *vkr* transcripts observed in males (Supplementary Fig. S3).

We then tried to perform a similar experiment in which mating occurred between males and females both silenced for *Anopheles* VKR. However, when both partners were injected, we found out that the efficiency of mating, even in the dsGFP controls, was very low (with number of laid eggs lower than 10), and we could not pursue the experiment.

### VKR is required for Anopheles immunity against *Plasmodium* infection

In addition to expression in gonads, VKR transcripts were also detected in the rest of the body^9^. Haemocytes and the fat body have been involved in insect immunity, but VKR function in these cells and organ was not already examined. The demonstration that haemocytes perfused from adult *A. coluzzii* females express VKR transcripts (Supplementary Fig. S2) allowed us to hypothesize that VKR could also be involved in *Anopheles* immunity. As a tyrosine kinase receptor, VKR is linked to different kinase pathways^13^ and possibly in *Anopheles* to the MAP Kinase and the JNK kinase, which are involved in the anti-*Plasmodium* response^24,25^.

**Figure 5:**
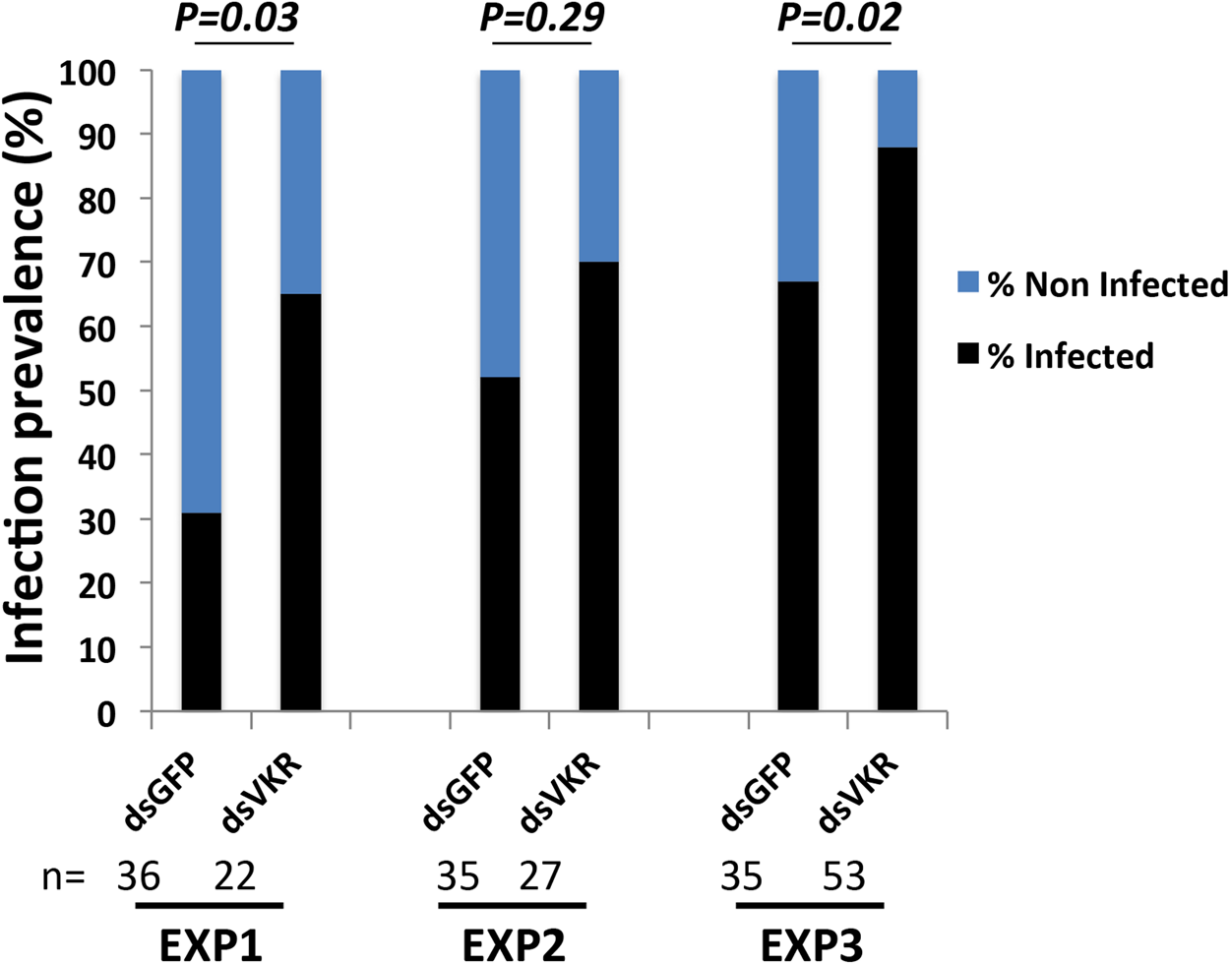
VKR is required for protective function against *Plasmodium* parasites. VKR restricts permissiveness of mosquitoes to *P*. *berghei* infection, measured as oocyst infection prevalence (proportion of infected mosquitoes). For each independent experiment, Chi-square p-values related to the proportion of infected mosquitoes (infection prevalence) between the two groups (dsGFP and dsVKR) are mentioned. Using the meta-analytical approach of Fisher to combine individual p-value related to infection prevalence from the three independent replicates, we found a statistically significant combined p-value (p = 0.0008).

To test a potential immune role for VKR, two groups of *A. coluzzii* females were injected with dsGFP (control) and dsVKR and 4 days post-injection, these two groups of females were infected with the rodent malaria parasite *Plasmodium berghei*. The midguts from the two groups of infected mosquitoes were dissected 8 days post-infection to assess their infection status. The results from three independent experiments showed an increase of infection prevalence in VKR-silenced background as compared to the dsGFP control group (Fig. 5). However no effect was observed on infection intensity (parasite load in infected midguts). These results reveal a novel function related to immunity for an insect VKR, in addition to its role in reproduction or in our case, in larval growth.

### VKR might be involved in the biosynthesis of 20E in *Anopheles* females

Then we focused our interest on particular steroid hormones, named ecdysteroids, which, in insects, play a major role during growth, development and reproduction^26^. The active form of the hormone is the 20-hydroxyecdysone (20E), which coordinates major developmental transitions^27,28^. We assessed whether VKR could be linked to the biosynthesis pathway of the 20E looking at the expression level of two P450 enzymes, CYP307A1 and CYP314A1, that are both involved in the biosynthesis of 20E in *Anopheles*^28^. We found that CYP314A1 expression level is reduced only in females silenced for VKR, but not in males (Supplementary Fig. S4). However CYP307A1 expression was not statistically affected by VKR silencing in both males and females.

## Discussion

In this study, we report the functional characterization of a member of the VKR family in the mosquito vector *A. coluzzii*. This RTK is encoded by a single gene located on the 3 R chromosome and sequence alignment with other insect VKRs revealed a high level of conservation of its functional domains. *Anopheles* VKR is, as the majority of the other VKR proteins, activated following the binding of arginine to the VFT extracellular domain and the structure of its TK domain is very similar to that of insulin receptors, an important characteristic of VKR molecules in terms of downstream kinase signalling. VKRs were first discovered in the helminth parasite *Schistosoma mansoni*, and they have been particularly studied in this organism for their function in reproduction. In this helminth, VKR is involved in larval growth processes but it is mainly required for the production of eggs by female worms^13^. The function of VKR in egg maturation was then confirmed in the mosquito vector *A. aegypti*^14^. In *Schistocerca gregaria*, VKR knockdown had significant effects on ovarian ecdysteroid levels and on the size of oocytes during the vitellogenic stage but it could not be concluded that VKR was essential for reproduction, since silencing did not affect fecundity or fertility in *S. gregaria*^15^. Therefore, it was important to analyse the function of these novel receptors in *Anopheles* in order to understand its importance in the biology and the physiology of this mosquito vector and to estimate the pertinence of such molecules, which are absent from the human host, as potential new vector targets.

**Figure 6:**
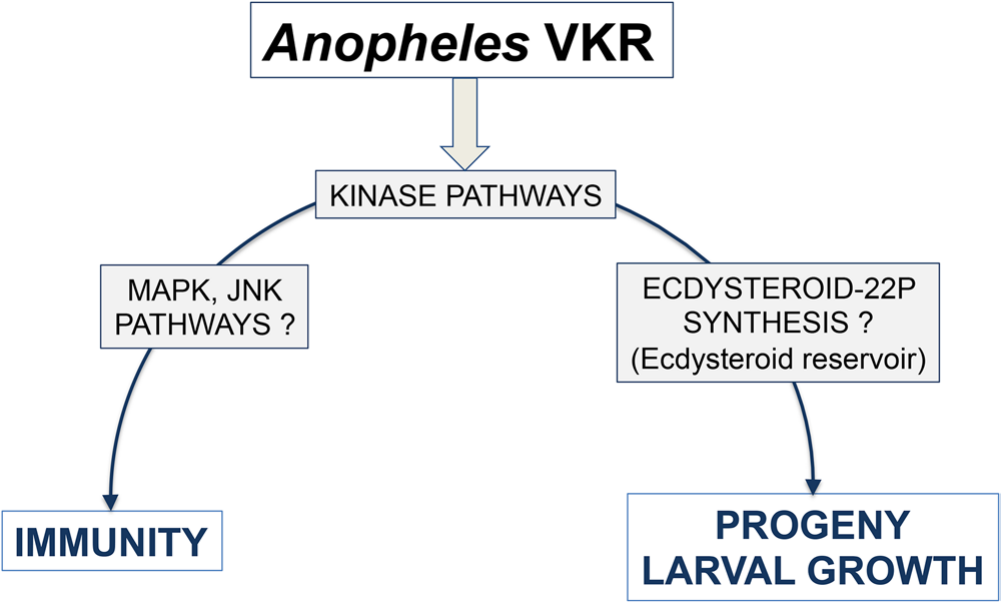
Proposed model for *Anopheles* VKR dual roles in both immunity and larval growth. As a tyrosine kinase receptor, activation of VKR in *A. coluzzii* would lead to activate kinase pathways, which are either involved in immunity (through potential activation of JNK and/or MAP pathways) or phosphorylate ecdysteroid (22P), which serve as a reservoir of ecdysteroids during the larval growth.

The functional characterization of *Anopheles* VKR has been performed using RNAi-mediated gene silencing assays in *A. coluzzii*. Our results showed that VKR displayed no function in *Anopheles* fecundity, as its silencing near affected the structure of the ovaries, nor the number of laid eggs per female (Fig. 3A–C and Supplementary Table S1). Nevertheless, decreasing the expression of the gene either in females (mothers) or in males (fathers) significantly affected the development of their larval progeny until the adult stage and with a stronger effect in male progeny. The weaker phenotype observed in female mothers as compared to males might be due to the difference of the silencing efficiency of *vkr* transcripts between females and males (Supplementary Fig. S3). Finally, we found out that VKR expressed in circulating haemocytes could play a protective role against *Plasmodium*, since dsVKR females displayed an increase of *Plasmodium* infection prevalence as compared to the control group (Fig. 5). The participation of VKR in the anti-*Plasmodium* phenotype still requires further work to identify the pathway(s) and the downstream immune factor(s), which are linked or regulated by VKR. However we could not exclude that VKR might be linked to the MAP Kinase and the JNK kinase, which are involved in the anti-*Plasmodium* response (model, Fig. 6). This dual role of VKR in both larval growth and immunity is reminiscent of the role of the *dorsal* regulatory cassette in *Drosophila*. *Dorsal* controls dorsal-ventral patterning during embryonic development, but during the adult stage the *Dorsal* regulatory mechanism is reused to regulate completely different genes involved in immunity^29,30^.

We then assessed whether VKR could be linked to the biosynthesis pathway of the 20E looking at the expression level of two P450 enzymes, CYP307A1 and CYP314A1, that are both involved in the biosynthesis of 20E in *Anopheles*^28^. As we found that only CYP314A1 expression level is affected in females silenced for VKR, but not in males (Supplementary Fig. S4), we could not exclude that VKR probably acts through another mechanism in *Anopheles*. For instance, it has been demonstrated that in insect ovaries, a phosphorylated form of ecdysteroid (ecdysteroid 22-phosphate), which is physiologically inactive, could serve as a “reservoir” in the ovaries that supplies active free ecdysteroids during embryonic development triggered by a phosphatase^31,32^. The formation of the phosphorylated ecdysteroid (ecdysteroid 22-P) requires the activity of an ecdysteroid kinase. As a kinase receptor, we could hypothesize that VKR would act upstream of this ecdysteroid kinase, or would belong to the kinase pathway that leads to produce the ecdysteroid 22-P (Fig. 6). Hence depleting VKR in *A. coluzzii* would impair the ecdycteroid kinase pathway and reduce the amount of ecdysteroid 22-P. Therefore in VKR silencing background, the reservoir of active free ecdysteroids further required during the larval growth might be reduced and could significantly impair the larval development.

The evolutionary history and function of VKRs might be different between the various organisms in which they have been found. In the case of two phylogenetically close organisms, *Aedes* and *Anopheles* mosquitoes, we did not observe the same function between *Aedes* VKR^14^ and *Anopheles* VKR, especially for the reproductive function. *Aedes* VKR displays an important function in fecundity, while *Anopheles* VKR does not. This might also be due to different ecological niches and different pressures acting on VKR genes. Nevertheless, as it was not checked in the study of AeVKR^14^, we could not exclude that the receptor also plays a role in males and in immunity in *Aedes* mosquitoes. Another study performed on the locust *S. gregaria* showed also different functions of SgVKR as compared to AeVKR^15^. This strengthens the hypothesis of a divergent evolution in VKR functions at least between these three insects (the locust and the two mosquitoes).

Altogether and consistent with the importance of its biological effects (Fig. 6), *Anopheles* VKR appears, through its dual role and the absence of ortholog in vertebrates, as an interesting novel insect target for controlling mosquito populations.

## Supplementary information

Supplementary informations
