# Supplementary material for: Dual role of the Anopheles coluzzii Venus Kinase Receptor in both larval growth and immunity
Source: Sci Rep. 2019 Mar 5;9:3615. doi: 10.1038/s41598-019-40407-x (PMC6401105; doi:10.1038/s41598-019-40407-x)
Supplement: Supplementary file 1 — Supplementary informations [file 41598_2019_40407_MOESM1_ESM.pdf]

Dual role of the *Anopheles coluzzii* Venus Kinase Receptor in both larval growth and immunity

Author list:

Nadège Gouignard, Floriane Cherrier[2], Emma Brito-Fravallo, Adrien Pain, Natalia Marta Zmarlak, Katia Cailliau, Corinne Genève, Kenneth D. Vernick, Colette Dissous*, Christian Mitri*

*: Correspondence: Colette Dissous, colette.dissous@pasteur-lille.fr; Christian Mitri, christian.mitri@pasteur.fr

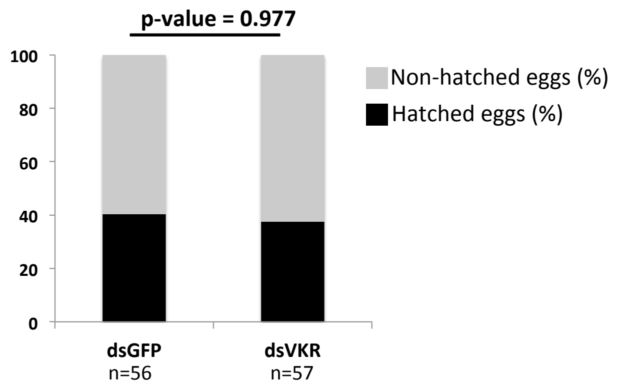

Supplementary Figure S1: No difference in the hatching rate between eggs from dsGFP and dsVKR females. Proportion of the egg hatching was assessed by counting the number of eggs that transformed into larvae over the total number of eggs put in water for both dsGFP and dsVKR group. n=Number of total eggs put in individual well for assessing the hatching. A Chi2 test was performed to analyse the difference in the hatching rate between the two groups (dsGFP and dsVKR).

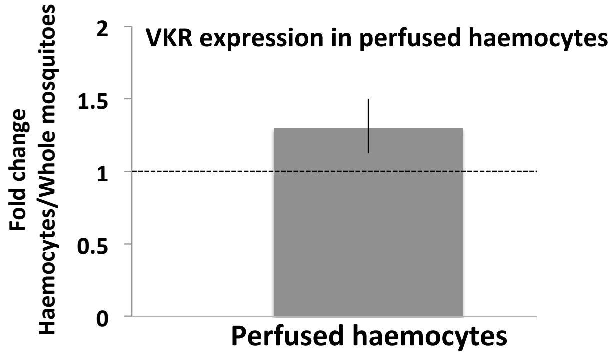

Supplementary Figure S2: Similar level of VKR expression level in perfused haemocytes as compared to whole *A. coluzzii* mosquitoes. Relative quantification of VKR gene expression in perfused *A. coluzzii* haemocytes, using expression of the ribosomal protein rps7 gene as the internal calibrator. The ratio of the normalized VKR expression in haemocytes *versus* whole mosquitoes was computed using triplicates from the same cDNA dilution. Error bars show median absolute deviation computed by permutation. The dotted line represents median expression level of VKR in whole *A. coluzzii* mosquitoes.

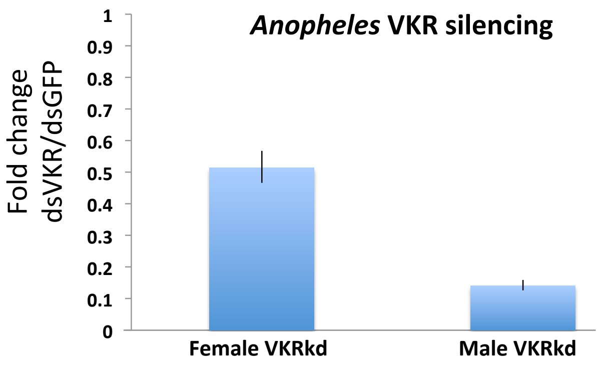

Supplementary Figure S3: Efficiency of VKR silencing in males and females. Relative quantification of VKR gene expression in RNAi-mediated VKR gene silencing experiments in males and females, using expression of the ribosomal protein rps7 gene as the internal calibrator. The ratio of the normalized VKR expression in VKRkd versus the GFP control was computed using triplicates from the same cDNA dilution. Error bars show median absolute deviation computed by permutation.

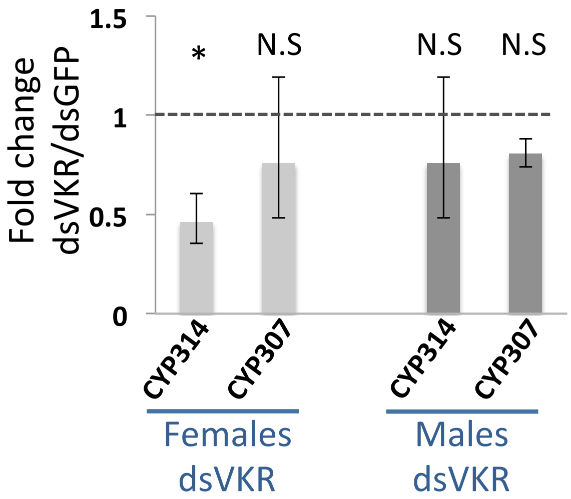

Supplementary Figure S4: VKR is not regulating CYP450 involved in the biosynthesis of 20-hydroxyecdysone in *A. coluzzii*. Relative quantification of CYP314 and CYP307 gene expression in RNAi-mediated VKR gene silencing experiments in males and females, using expression of the ribosomal protein rps7 gene as the internal calibrator. The ratio of the normalized gene of interest versus the GFP control was computed using triplicates from the same cDNA dilution. Error bars show median absolute deviation computed by permutation. The dotted line represents median expression level of CYP314 and CYP307 in male or female mosquitoes. Star (*= $p<0.05$) shows statistically significant p-value related to deltaCt distribution between dsGFP and dsVKR. NS= Non statistically significant p-value.

dsRNA injection in males	dsGFP	dsVKR	*P-value (Chi-test)*
Nb of females (mothers)	40	40	
Nb of laid eggs (Rep1)	221	147	*0.1266*
Nb of laid eggs (Rep2)	154	180	*0.6157*

Supplementary Table S1: VKR silencing in males has no effect on the number (Nb) of laid eggs/female mothers. In two independent experiments, VKR silencing in males has no effect on the number (Nb) of laid eggs/female mothers (p-value>0.05). The number of female mothers for each group (dsGFP and dsVKR) was the same (40 females) for each biological replicate.

**T7-GFP-F**	**GAATTGTAATACGACTCACTATAGGGCATGGTGAGCAAGGGCGAG**
**T7-GFP-R**	**GAATTGTAATACGACTCACTATAGGGCTTACTTGTACAGCTCGTC**
**T7-VKR-F**	**TAATACGACTCACTATAGG ATTACATCCCGGGCGAGATCCG**
**T7-VKR-R**	**TAATACGACTCACTATAGG GACGGCGTCCAGTGAAACACG**
**rpS7q-F**	**CACCGCCGTGTACGATGCCA**
**rpS7q-R**	**ATGGTGGTCTGCTGGTTCTT**
**VKR-q-F (Verif)**	**AACCGGTCTACACGGTGATGG**
**VKR-q-R (Verif)**	**CACCGGCAGCATGCCGCGCCG**
**CYP307-q-F**	**CAACAACCTCGACCTGATCC**
**CYP307-q-R**	**CTTGGCGAACAGTGCTTCC**
**CYP314-q-F**	**TCCGGCATCATTGACCTCAT**
**CYP314-q-R**	**AATCTCGCGCTGATACTCCA**

Supplementary Table S2: Primer sequences. Sequence of the primers used for the synthesis of the double-stranded RNA (GFP or VKR) are preceded by the T7 sequence (underlined) and primers used for knockdown verification are labelled as (Verif). rpS7 primers (F) and (R) were used for the internal calibration for each qPCR experiment. The sequence primers of CYP307 and CYP314 were used to measure by qPCR the expression of these two genes under dsGFP and dsVKR backgrounds.

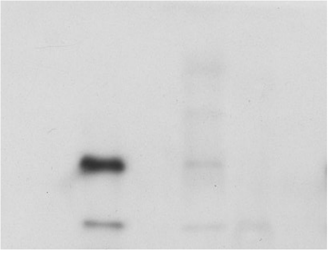

Complementary information 1: Original blot related to the detection of phospho-tyrosine (using the anti-phosphotyrosine).

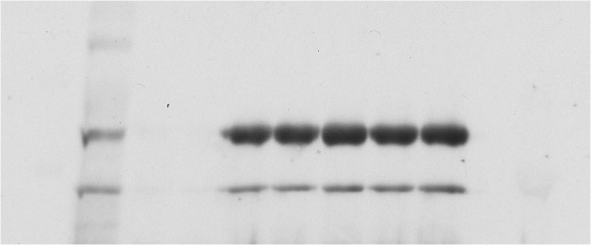

Complementary information 2: Original blot related to the detection of the V5-tagged region of VKR (using the anti-V5).
